# Global area strain is a sensitive marker of subendocardial damage in adults after optimal repair of aortic coarctation: three-dimensional speckle-tracking echocardiography data

**DOI:** 10.1007/s00380-016-0803-4

**Published:** 2016-02-03

**Authors:** Ewa Kowalik, Mirosław Kowalski, Anna Klisiewicz, Piotr Hoffman

**Affiliations:** Department of Congenital Heart Disease, Institute of Cardiology, Alpejska 42, 04-628 Warsaw, Poland

**Keywords:** Aortic coarctation, Left ventricular function, Speckle-tracking echocardiography, Three-dimensional imaging, Area strain

## Abstract

Aortic coarctation (CoA) in adults is associated with reduced survival. Despite successful repair, some unfavorable changes in the left ventricular (LV) myocardial function are reported. Three-dimensional speckle-tracking imaging (3D-STE) is a novel method that allows to assess regional myocardial function in all directions simultaneously and to calculate global area strain which integrates longitudinal and circumferential deformation. The aim of our study was to assess whether 3-D STE provides any new characteristics of LV deformation in patients with optimal CoA repair. Adults after CoA correction underwent transthoracic echocardiographic examinations. Patients with significant concomitant lesions were ruled out. Global longitudinal strain (GLS), global circumferential strain (GCS), global area strain (GAS), and global radial strain (GRS) were assessed using 3D-STE (Echopac Software, GE). The data were compared with those obtained from healthy subjects. 26 adults (9F/17M; mean age 24.4 years) with repaired CoA were studied. Despite preserved LVEFs, patients with repaired CoA had decreased GAS compared with controls (−28.8 vs. −31.7 %; *p* = 0.007). No differences between patients and healthy subjects in terms of GLS, GCS and GRS were observed. We found a significant correlation between mean blood pressure and GAS (*R* = 0.39; *p* < 0.05). No significant influence of age at repair, CoA correction method or LV mass on three-dimensional deformation was observed. Summarizing, global area strain derived from 3D-STE may be a sensitive indicator of subclinical LV dysfunction in patients after optimal repair of CoA. Mean blood pressure, but not age at correction seems to determine LV deformation.

## Introduction

Coarctation of the aorta (CoA) is not a localized lesion of the vessel, but a diffused cardiovascular disease. Despite successful surgical or catheter interventional treatment, CoA is known to be associated with high long-term morbidity and shortened life expectancy [1, 2]. In patients treated due to CoA some unfavorable changes in the left ventricular (LV) myocardial function are observed. Data on LV regional function in patients with optimal CoA repair are scarce and limited to two-dimensional imaging [3, 4]. Three-dimensional speckle-tracking echocardiography (3D-STE) is a novel method that enables quantitative analysis of regional myocardial function of all LV segments in all dimensions simultaneously. The latter technique can also offer a new deformation parameter, area strain, that quantifies endocardial area change and integrates both longitudinal and circumferential deformation [5]. Global area strain (GAS) has been proved to detect early LV systolic dysfunction in athletes, in patients after heart transplantation or after anthracycline therapy, as well as in patients with diabetes and autoimmune disorders [6–10]. Of note, 3D-STE turned out to be less time consuming than 2D in respect to the acquisition and analysis time [11, 12], thus the former has a greater chance to be routinely used in the clinical practice. The aim of our study was to assess whether 3D-STE provides any new information on myocardial deformation in subjects with optimal repair of CoA.

## Methods

### Study population

We studied 26 consecutive adult patients after surgical or percutaneous CoA correction who were referred to our outpatient clinic for a routine check-up. All patients had preserved LV ejection fraction (EF). The exclusion criteria for the study were: (1) age >50 years, (2) CoA repair <6 months before the study, (3) recurring or residual CoA that require intervention, (4) significant concomitant congenital cardiac defects (patients with bicuspid aortic valve were included unless they presented aortic stenosis of any degree and/or more than mild aortic regurgitation), (5) poorly controlled systemic arterial hypertension in follow-up at our center (systolic BP ≥ 140 mmHg and/or diastolic BP ≥ 90 mmHg), (6) any evidence of coronary heart disease, (7) arrhythmia at the echocardiographic study, (8) asymptomatic left ventricular dysfunction (LV EF < 55 %) or congestive heart failure. Echocardiographic data obtained in CoA patients were compared with those of a control group of 18 healthy volunteers who did not have any structural or functional cardiovascular abnormalities, nor did they take any medications. The study was approved by the local ethics committee and each patient signed informed written consent.

### Standard echocardiographic imaging and aortic elastic parameters

Standard transthoracic echocardiographic examinations were performed using commercially available equipment (Vivid 9, GE Vingmed Ultrasound, Horten, Norway) with a matrix probe M3S. Left ventricular end-diastolic (LVEDD) and end-systolic diameters, as well as intraventricular septum (IVS) and posterior wall (PW) thickness were obtained according to ASE guidelines [13]. Relative wall thickness (RWT) was calculated by the formula (2xPW)/LVEDD [14]. Left ventricular ejection fraction (EF) was calculated using the biplane Simpson’s method. Left ventricular mass (LVM) was measured according to the Devereux formula [15] and indexed for body surface area. Systolic and diastolic aortic diameters were measured 3 cm above the aortic valve by 2-D guided M-mode echocardiography at the left parasternal long-axis view: aortic systolic diameter (AoS) at the time of opening of the aortic valve and diastolic diameter (AoD) at the *R* wave of electrocardiogram. The aortic arch and descending aorta diameters were measured in the suprasternal view. Maximal and mean pressure gradients across the coarctation site were assessed using continuous wave Doppler recordings in the same view. Blood pressure (BP) measurements at the right arm were obtained using a cuff sphygmomanometer (Omron M1, Omron Healthcare Co, Kyoto, Japan). The average of three successive readings was taken into account. Central aortic pulse pressure (PP) was defined as a difference between systolic (SBP) and diastolic blood pressure. Subsequently, we calculated three indices of aortic elastic properties [16]:

$$\begin{aligned} & {\text{Aortic strain}}\left( \% \right) = 100 \times \left( {{\text{AoS}} - {\text{AoD}}} \right) / {\text{AoD}} \\ & {\text{Aortic distensibility}}\left( {{\text{cm}}^{ 2} / {\text{dyn/1}}0^{ 6} } \right) = 2\times \left( {{\text{AoS}} - {\text{AoD}}} \right)/\left( {\text{AoD}} \times {\text{ PP}} \right) \\ & {\text{Aortic stiffness index}} = \left[ {{ \ln }\left( {\text{SBP/DBP}} \right)} \right]/\left[ {\left( {{\text{AoS}} - {\text{AoD}}} \right)/{\text{AoD}}} \right] \\ \end{aligned}$$

### 3D-STE analysis

**Fig. 1:**
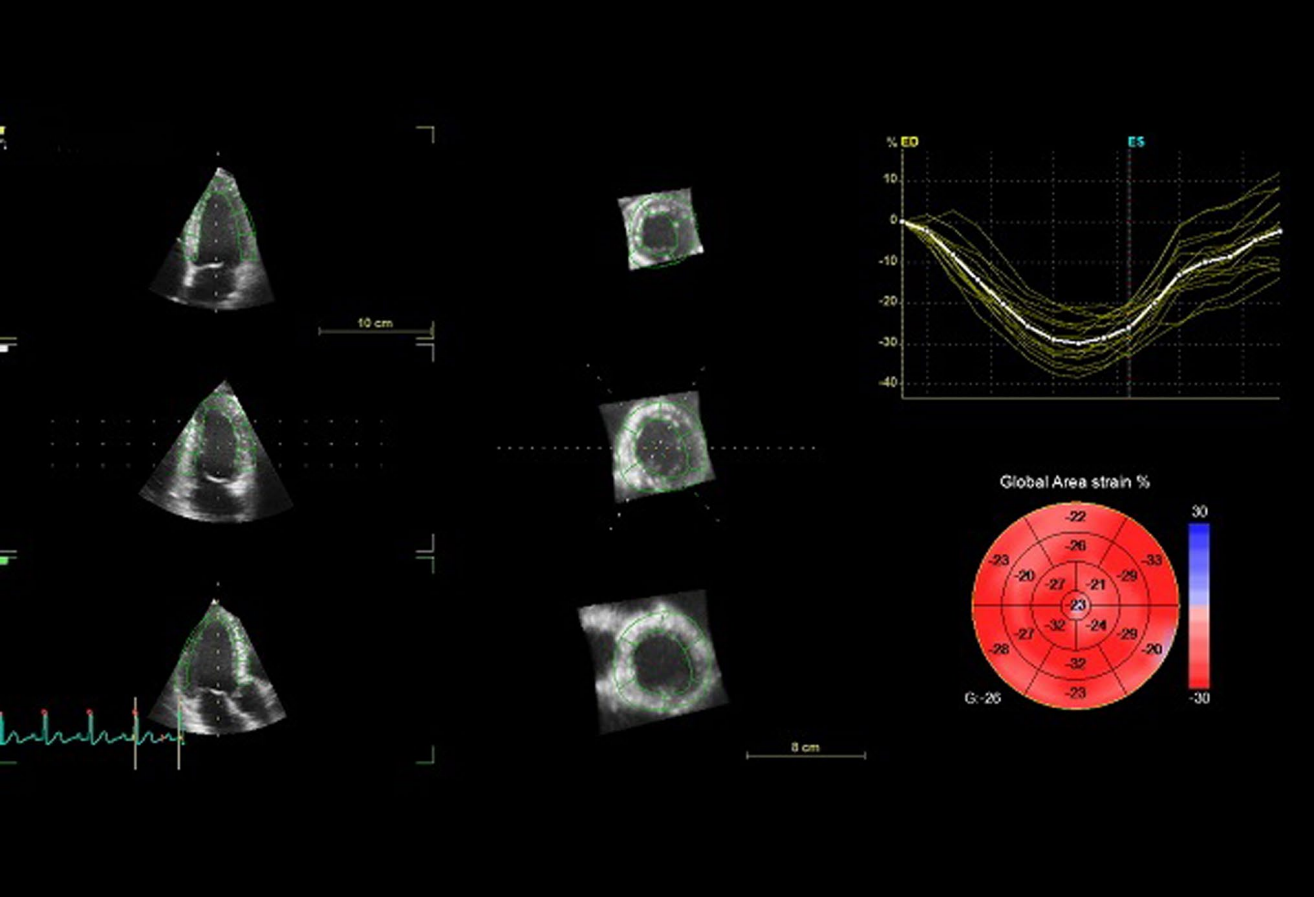
An example of 3D-STE analysis

During the same examination 3D full-volume data sets were acquired from the apical view using the matrix-array transducer 4V-D. Four to six ECG-gated beats were recorded during end-expiratory breath hold to create LV full volume. The volume size and depth were individually adapted and the mean temporal resolution was 25.5 vol/s. 3D data were analyzed offline using EchoPac software (version 113, GE Vingmed Ultrasound). The software automatically detects the LV endocardial border in 3D, and, after manual adjustment, calculates the LV volumes, cardiac output, stroke volume, EF and LV sphericity index. Next, the definition of the epicardial boundary allows LV mass and 3D global myocardial deformation parameters [longitudinal (GLS), circumferential (GCS), radial (GRS) and area (GAS) strain] to be measured. The area strain is defined as the percentage change in the endocardial area at LV end-systole from its original area at end-diastole [11]. All global strains are weighted averages of regional values from 17 myocardial segments of LV (Fig. 1). Rejected segments (determined automatically by the software) were not taken into account during the calculations of the global strain values and more than three rejected segments in one patient resulted in exclusion from any further analysis. All parameters were compared to those obtained from the healthy subjects in the control group.

### Reproducibility

Intra-observer and inter-observer reproducibility was assessed in 15 randomly chosen subjects. It was expressed as percentage of error, derived as the absolute difference between 2 sets of measurements divided by the mean of the observations and using intraclass correlation coefficients. Intra-observer measures were performed at least 1 month apart. To calculate inter-observer variability the second experienced observer (M.K.), who was blinded to the first observer’s findings, analyzed 3D-STE data sets.

## Statistics

Results, unless stated otherwise, are presented as the mean value ± SD. Normal distribution of variables was checked using the Kolmogorow–Smirnov test. The comparisons of mean differences between the groups were made using unpaired Student’s t test and the linear Pearson correlation was used to indicate the strength of a relationship between GAS and other parameters taken into account. In the multivariate regression analysis, the backward elimination method with t tests was used to select variables. A confidence level of *p* < 0.05 was considered statistically significant.

## Results

### Clinical data

Twenty-six patients (9 women and 17 men), mean age 24.4 ± 6.7 years (range 18–41 years) after surgical (*n* = 21) or percutaneous (*n* = 5) CoA correction were studied. CoA repair had been performed at a mean age of 8.6 ± 12.1 years (range 0–40 years). In half of the study group bicuspid aortic valve (with no stenosis and only trivial regurgitation) was diagnosed and 58 % of patients were on antihypertensive treatment. No significant differences were observed between patients and controls with respect to age, gender and heart rate. Patients had significantly higher systolic and mean blood pressure. Clinical characteristics of the study group are given in Table 1.

**Table 1:** Demographics of the study population

	Coarctation patients	Healthy controls	*p* value
Mean age (year)	24.4 ± 6.7	27.2 ± 6.7	NS
Men [*n* (%)]	17 (65)	8 (44)	NS
BMI	23.8 ± 3.5	23.5 ± 2.9	NS
Heart rate (beats/min)	59 ± 10	63 ± 9	NS
Systolic blood pressure (mmHg)	129 ± 12	119 ± 9	0.003
Diastolic blood pressure (mmHg)	80 ± 10	76 ± 6	NS
Mean blood pressure (mmHg)	96 ± 10	90 ± 6	0.016
Bicuspid aortic valve [*n* (%)]	13 (50)		
Mean age at time of intervention (year)	8.6 ± 12.1		
Mean time from repair (year)	15.8 ± 8.7		
*Treatment history*			
Endovascular stenting [*n* (%)]	5 (19)		
Prosthetic patch [*n* (%)]	4 (15)		
End-to-end repair [*n* (%)]	7 (27)		
Waldhausen operation [*n* (%)]	10 (38)		
Antihypertensive treatment [*n* (%)]	15 (58)		
ACEI/ARB [*n* (%)]	9 (35)		
CBB [*n* (%)]	11 (42)		
BB [*n* (%)]	2 (8)		
Diuretics [*n* (%)]	3 (12)		

*BMI* body mass index, *ACEI* angiotensin converting enzyme inhibitor, *ARB* angiotensin receptor blocker, *CBB* calcium channel blocker, *BB* beta blocker

### 2D echocardiographic data

Standard echocardiographic parameters in patients and in controls are presented in Table 2. As expected, LV diameters and EF were in normal range and similar between the groups. CoA patients presented an increased LV wall thickness and LV mass comparing to controls. Aortic coarctation was associated with increased aortic stiffness index values. A decrease in aortic distensibility and aortic strain was also observed across the CoA group. 2D echocardiographic examination also revealed that aortic arch and descending aorta were narrower in CoA patients. The mean/peak gradient across coarctation site assessed in patients was 11.2/24.4 mmHg.

**Table 2:** Standard echocardiographic parameters

	Coarctation patients	Healthy controls	*p* value
IVS (mm)	9.8 ± 1.6	8.7 ± 1.6	0.02
PW (mm)	9.9 ± 1.2	8.2 ± 0.9	<0.001
LVED (mm)	50.2 ± 5.1	50.5 ± 3.6	NS
LVSD (mm)	29.9 ± 4.3	31.4 ± 3.4	NS
EF (%)	66 ± 4.7	66.9 ± 5.2	NS
RWT	0.4 ± 0.06	0.33 ± 0.04	<0.001
LVM (g)	183.1 ± 52.1	151.8 ± 35.7	0.02
LVM index	98.4 ± 23.2	81.1 ± 13.3	0.003
MAPSE (mm)	14.5 ± 1.6	16.1 ± 1.9	0.01
E/A ratio	1.86 ± 0.73	1.85 ± 0.73	NS
*Aortic elastic properties*			
Aortic systolic diameter (mm)	30.4 ± 7.5	27.6 ± 3.0	NS
Aortic diastolic diameter (mm)	27.8 ± 7.6	24.4 ± 3.2	0.03
Aortic strain (%)	10.2 ± 5.7	14.3 ± 6.7	0.04
Aortic distensibility	3.2 ± 1.6	6.4 ± 3.6	0.003
Aortic stiffness index	7.1 ± 5.4	4.0 ± 2.8	0.02
Aortic arch (mm)	21.0 ± 3.8	23.0 ± 2.1	0.04
Descending aorta (mm)	14.4 ± 2.4	17.3 ± 2.1	<0.001
Mean gradient across coarctation site (mmHg)	11.2 ± 8		
Peak gradient across coarctation site (mmHg)	28.4 ± 14		

*IVS* interventricular septum, *PW* posterior wall, *LVED* left ventricular end-diastolic diameter, *LVSD* left ventricular systolic diameter, *EF* ejection fraction, *RWT* relative wall thickness, *LVM* left ventricular mass

### 3D echocardiographic data

#### Feasibility

Out of all LV segments analyzed 9.1 % were not interpretable and excluded from the analysis of global deformation. The most frequently rejected segments were these located in the posterior wall (23.5 % of all nonanalyzable segments). An average volume rate for 3D acquisition was 25.5 ± 9.2 vol/s.

#### 3D analysis

3D echocardiographic examination showed that LV volumes and EF were similar in both groups (Table 3). Similarly to the 2D echocardiography, LV mass was significantly higher in patients. Moreover, the 3D LV mass values were higher than the 2D-derived ones, which is consistent with the results of previous studies comparing 2D and 3D echocardiographic parameters [17]. All parameters of 3D LV deformation tended to be lower in CoA group, however, only GAS differed significantly in the CoA group. No differences in LV deformation data were found between patients with bicuspid aortic valves compared with tricuspid ones. Nor did the method of CoA correction influenced the LV deformation values. Similarly, there were no differences in 3D deformation values between patients with hypertensive treatment and patients with no medications, although higher LV mass and lower diastolic RR in individuals on antihypertensive medications were observed (Table 4). Both subgroups of patients differ significantly from the control group in terms of GAS values (*p* < 0.05).

**Table 3:** Real-time three-dimensional echocardiographic assessment

	Coarctation patients	Healthy controls	*p* value
LV EDV (mL)	107.8 ± 33.6	113.5 ± 23.3	NS
LV ESV (mL)	41.3 ± 17.6	44.2 ± 12.0	NS
SV (mL)	65.8 ± 17.4	69.4 ± 13.6	NS
CO (L/min)	4.13 ± 1.4	4.68 ± 1.3	NS
LV EF (%)	61.8 ± 6.9	61.4 ± 4.8	NS
Sphericity index	0.45 ± 0.09	0.44 ± 0.09	NS
LVM (g)	137.6 ± 22.4	124.3 ± 16.5	0.02
LVM index	75.423 ± 11.9	67.944 ± 8.6	0.01
GLS (%)	−16.6 ± 3.8	−18.4 ± 2.5	NS
GCS (%)	−16.7 ± 2.8	−17.5 ± 2.4	NS
GAS (%)	−28.8 ± 4.1	−31.7 ± 2.7	0.007
GRS (%)	47.1 ± 10.1	51.3 ± 6.5	NS

*EDV* end-diastolic volume, *ESV* end-systolic volume, *SV* stroke volume, *CO* cardiac output, *LVM* left ventricular mass, *GLS* global longitudinal strain, *GCS* global circumferential strain, *GAS* global area strain, *GRS* global radial strain

**Table 4:** Selected clinical parameters and echocardiographic indices in coarctation patients with no hypertension and in coarctation patients on antihypertensive medications

	CoA pts with no hypertension (*n* = 11)	CoA pts on antihypertensive medications (*n* = 15)	*p* value
Mean age (year)	25.5 ± 6.4	23.5 ± 7.0	NS
Systolic blood pressure (mmHg)	129.7 ± 11.4	128.3. ± 12.3	NS
Diastolic blood pressure (mmHg)	84.8 ± 7.5	76.3 ± 10.1	0.01
LVM index	87.5 ± 14.5	106.4 ± 43.8	0.01
GLS (%)	−16.0 ± 4.4	−17.0 ± 3.4	NS
GCS (%)	−16.0 ± 1.6	−17.2 ± 3.4	NS
GAS (%)	−27.9 ± 4.0	−29.4 ± 4.1	NS
GRS (%)	44.9 ± 8.8	48.7 ± 11.0	NS

*CoA* aortic coarctation, *LVM* left ventricular mass, *GLS* global longitudinal strain, *GCS* global circumferential strain, *GAS* global area strain, *GRS* global radial strain

**Fig. 2:**
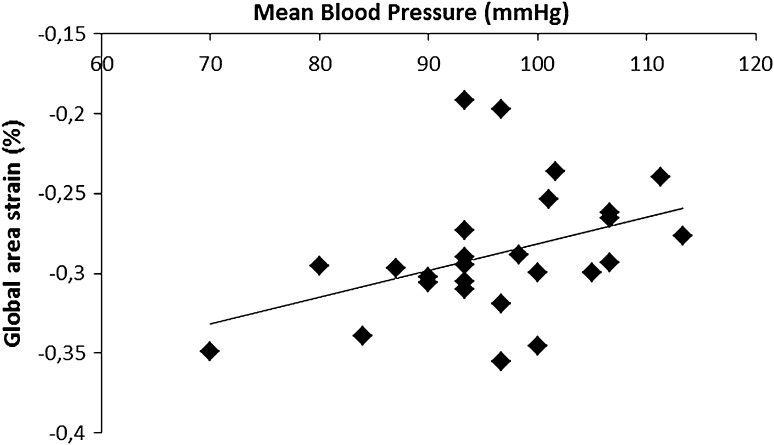
The correlation between mean BP and GAS in CoA patients (*r* = 0.39; *p* < 0.05)

Among clinical and echocardiographic parameters characterizing patients with CoA only mean blood pressure correlated significantly with global area strain (*R* = 0.39; *p* < 0.05), Fig. 2. Neither age at correction nor LV mass nor aortic elasticity indices correlated with the degree of LV deformation assessed by 3D-STE. Similarly, the multivariate regression showed that mean BP was the only parameter independently associated with LV GAS (cumulative *R* ^2^ = 0.62; *p* = 0.002).

#### Reproducibility

**Fig. 3:**
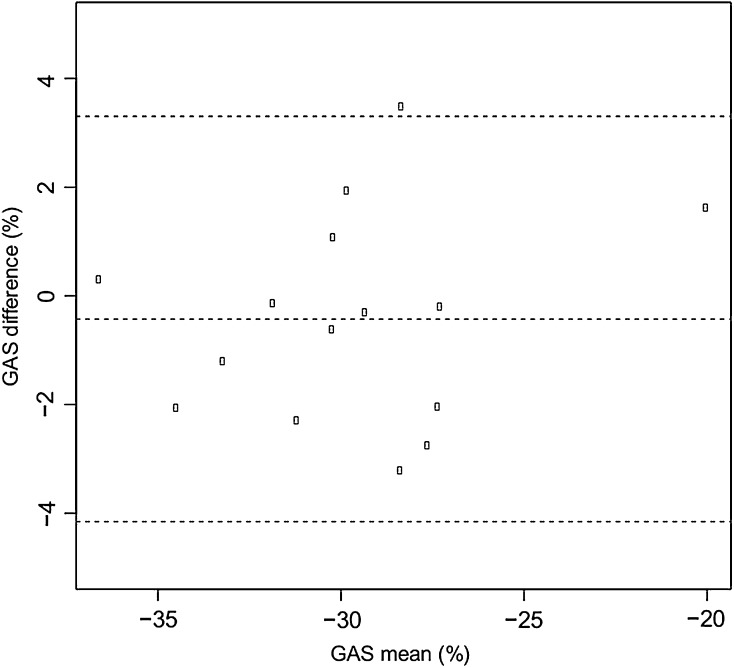
Bland–Altman plot for intra-observer difference of the global area strain

**Fig. 4:**
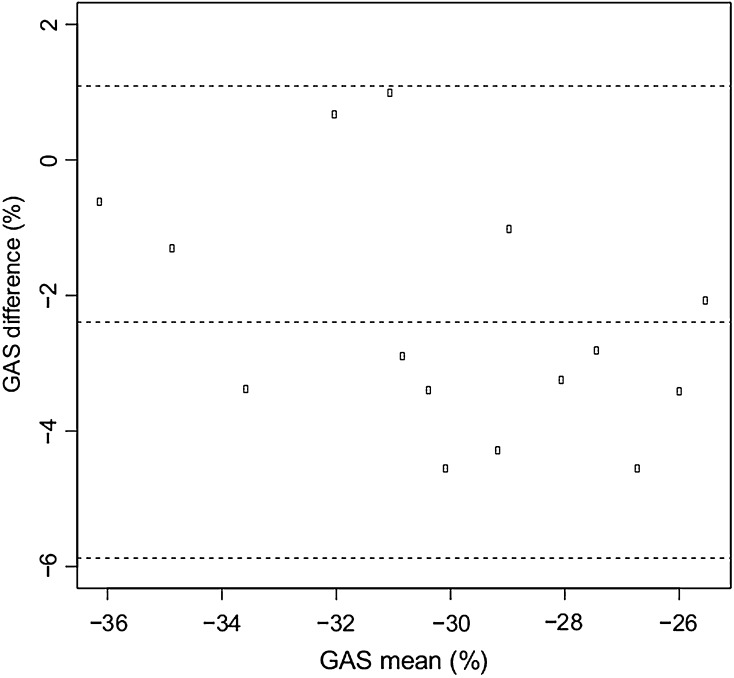
Bland–Altman plot for inter-observer difference of the global area strain

Intra- and inter-observer variability for global area strain was 5.4 % (ICCs 0.884) and 9 % (ICCs 0.641). The values are comparable to those reported for GAS in normal adults [11] and patients with other heart diseases [18]. Bland–Altman plots of the GAS intra- and inter-observer differences are presented in Figs. 3, 4.

## Discussion

It was the first study that examined 3D LV deformation from echocardiography in adults after optimal CoA repair. The important new finding is the subtle LV myocardial dysfunction as assessed by global area strain. Area strain reflects the change in the endocardial surface from its original dimensions at end-diastole and it integrates both longitudinal and circumferential deformations of the LV. We speculate that reduced GAS values can reflect subclinical microvascular abnormalities in the population analyzed. In the present study the remaining deformation parameters (longitudinal, circumferential and radial strain) did not differ significantly, which corresponds with optimal clinical outcome of CoA repair. Our results are in agreement with those by Cook et al. who studied a similar group of adults with repaired CoA. Using non-invasive angiography in combination with CMR the authors demonstrated subendocardial perfusion abnormalities despite a lack of LV hypertrophy, epicardial coronary artery disease or recoarctation [19]. Thus, it seems that the novel 3D deformation parameter, global area strain, might become an early non-invasive indicator of subclinical endocardial dysfunction. In contrast, Kutty et al. [20] using CMR based feature-tracking showed no differences between the COA group with normal LV mass and normal controls in terms of GLS and GCS. However, lower GRS values were observed in the patients group. In our opinion these results should be interpreted with caution due to the significant difference in the mean age of compared groups (37.1 years in controls vs. 23.3 years in CoA patients with normal LV mass, *p* value not given) as the LV deformation values proved to be age-related [21, 22].

The subendocardial layer of the myocardial fibers is more vulnerable to functional impairment due to direct impact of intraventricular blood pressure and the unfavorable coronary flow in this area. Our analysis showed that the reduction of GAS was associated with an increased pressure overload. The results are consistent in this field with our previous observation that a higher degree of narrowing across the coarctation site leads to greater reductions in local 2D deformation indices for the midsegment of the LV anterior wall [4]. Similar findings regarding the association between GAS and mean BP have also been reported previously in subjects with high-normal blood pressure [12] and native hypertensive patients [23]. However, we observed decreased global area strain values in patients after CoA repair on hypertensive treatment as well as in patients with no hypertension. Thus, the changes found in GAS cannot be explained only by the presence of hypertension.

In contrast to previous data on patients with aortic valvular disease [24] or hypertension [23], no association between global deformation parameters and the degree of LV hypertrophy (LVM or RWT) were found in the present study. However, the analyzed population included patients with only slightly increased LV mass and no clear-cut LV hypertrophy.

The age at correction did not have an impact on the values of 3D deformation indices in patients with optimal CoA repair, neither was such a relationship found in our previous study in adults where 2D deformation indices were used [4]. On the other hand, Di Salvo et al. [3] showed a significant correlation between 2D strain rate and age at correction in children after successful CoA correction. This difference might result from a much younger population analyzed earlier after repair in their study.

Our findings regarding aortic elastic properties are consistent with those reported in previous studies, which showed that aortic strain, distensibility and stiffness index of the ascending aorta remained abnormal despite successful CoA repair [16, 25]. However, the aortopathy did not go along with a significant correlation between these parameters and indices of 3D LV deformation. It is noteworthy that Di Salvo et al. [3] confirmed the relationship between 2D deformation (strain rate) and aortic stiffness index in children after CoA repair. The possible explanation might be a different study population as mentioned above. In addition, the method of deformation analysis (2D longitudinal strain rate derived from septum and LV lateral wall) could impact the results.

Summarizing, our study provides new insights into the LV regional function in patients after optimal CoA repair and the potential explanation of higher rate of cardiac events and reduced life expectancy in this population. The decreased GAS might be an early indicator of late cardiovascular complications and 3D-STE seems to be useful in risk stratification. Thus, further studies will help to define the clinical benefit of 3D deformation analysis.

## Limitations

The major limitation of our study relates to the small sample size mainly due to restrictive inclusion criteria as we decided to assess patients after optimal CoA repair with no significant comorbidities. Different techniques of CoA correction should also be pointed out. Another issue corresponds to the influence of LV imaging quality on deformation analysis. The feasibility of 3D-STE was high in the present study, however, our patients were relatively young and they presented no substantial myocardial hypertrophy. In the study we analyzed only global deformation parameters. The analysis of regional function of 17 LV segments might provide more information, however, such an approach is much more time consuming and hard to apply in every day practice. Finally, the limitations of the 3D-STE analyses are different vendor’s algorithms with poor inter-vendor reproducibility [26] that makes the results of the studies difficult to compare.

## Conclusions

Global area strain derived from three-dimensional speckle-tracking echocardiography detects LV myocardial damage in a subclinical stage in patients after optimal repair of CoA. Mean blood pressure, but not the age at correction or techniques of CoA repair impacts LV deformation.
